# Editorial: Microstructures and Mechanical Properties of Cement-Based Composites

**DOI:** 10.3390/ma16206636

**Published:** 2023-10-11

**Authors:** Lik-Ho Tam, Ao Zhou, Zechuan Yu, Chao Wu

**Affiliations:** 1 School of Transportation Science and Engineering, Beihang University, Beijing 100191, China; leo_tam@buaa.edu.cn; 2 School of Civil and Environmental Engineering, Harbin Institute of Technology, Shenzhen 518055, China; zhouao@hit.edu.cn; 3 School of Civil Engineering and Architecture, Wuhan University of Technology, Wuhan 430070, China; zecyui@whut.edu.cn; 4 Department of Civil and Environmental Engineering, Imperial College London, South Kensington, London SW7 2AZ, UK

In recent years, with the fast development of the technology and the economy associated with the growth of the global population, the construction of economical, sustainable, and eco-friendly infrastructures with improved ductility, resistance to external elements, and durability has increased the need for the development of high-performance construction materials. Accordingly, constituents including fibers, waste plastics, biominerals, and industrial wastes have been increasingly used as the reinforcing and/or replacing phases to develop the cement-based composites. The addition of these constituents leads to changes in the microstructures of cement-based composites. The synergistic effect between the added constituents and cement matrix leads to the enhanced properties of cement-based composites compared to conventional construction materials. Meanwhile, the addition of recyclable, readily available, and low-cost constituents for manufacturing cement-based composites is important to achieving a circular economy and sustainable development. Therefore, cement-based composites have increasingly been used in the construction and strengthening of transportation and civil infrastructures. This Special Issue has recently been organized to collect scientific and engineering advances in the investigation of the microstructures and properties of the cement-based composites, and it includes seven articles.

In order to facilitate the applications of cement-based composites, we require an understanding of composite microstructures for achieving advanced mechanical properties. Zhang et al. [1] showed that by increasing either the curing time or the cement–sand ratio, it was possible to increase the bond strength and bond number between sand particles, which enhanced peak strength, residual strength, and pre-peak stiffness of cemented sand, and the failure pattern changed from homogenous deformation to distinct shear band at the bottom of the samples. Comparatively, by decreasing the initial void ratio, similar enhancement was observed, but the residual strength and failure pattern were insensitive. These results provide valuable references for sand stabilization via cementation. In another investigation into high-performance polypropylene fiber-reinforced concrete (HPFRC) under the effect of high temperature conditions of 200, 400, and 600 °C, Kaczmarczyk et al. [2] reported that at high temperatures, the enhanced properties of HPFRC were not obvious due to the complete melting of the polypropylene fibers. However, the melting of polypropylene fibers could provide free space to reduce pore pressure, which caused a delay in the development of micro-cracks in the HPFRC structure, and the little change in the HPFRC structure could increase the heat resistance of concretes.

Although the cement-based composites possess enhanced properties, the manufacturing process consumes large amount of natural resources and energy and is expensive. In order to conserve natural resources, reduce CO_2_ emissions, and reduce production costs, extensive instigations have been carried out to apply cement replacement materials and recycled solid wastes to producing the composites. Al-Hashem et al. [3] used metakaolin (MK) that is locally available at negligible cost to partially replace cement by 5, 10, 15, and 20 wt% and found that there were a decreasing workability, an increasing compressive strength, and an increasing water absorption percentage of concrete upon increasing the percentage of MK. Specifically, the concrete with 20 wt% MK showed a maximum loss of workability of almost 60%, a maximum increase of 33.43% in compressive strength after 90-day curing, and a maximum water absorption increase of 20.08%. Meanwhile, via immersion in 2% sulfuric acid solution, the compressive strength of concrete samples showed no significant decrease after 56 days but a considerable decrease after 90 days compared to samples at the same age but without acid attack. Ground-granulated blast furnace slag (GGBFS) is a solid waste largely produced in the iron industry. Zhang et al. [4] mixed it with alkaline solutions (NaOH or Na_2_SiO_4_ solution) and magnesium salts (MgCl_2_·6H_2_O and MgSO_4_) to prepare the GGBFS-based geopolymers. It is reported that the magnesium salt-free Na_2_SiO_4_-activatied geopolymer exhibited a much higher 28 d compressive strength (63.5 MPa) than the salt-free NaOH-activated geopolymer (31.4 MPa), with the former mainly containing an amorphous phase (C-(A)-S-H gel) and the latter containing numerous crystals. Meanwhile, both MgCl_2_·6H_2_O and MgSO_4_ degraded the alkali-activated GGBFS-based geopolymers. Compared to the salt-free geopolymer, the Na_2_SiO_4_-activated geopolymer containing 8.5 wt% MgCl_2_·6H_2_O exhibited a lower 28 d compressive strength (30.1 MPa) and a more loosely bound geopolymer matrix, and the Na_2_SiO_4_-activated geopolymer with 9.0 wt% MgSiO_4_ exhibited a lower compressive strength of 42.8 MPa. Red sandstone (RS) is locally abundant in nature or found in the residue derived from excavation during road construction in certain regions. Kong et al. [5] partially substituted cement (OPC) via RS and reported that RS enhanced the flowability of RS-OPC composite but lowered the mechanical strength, with a maximum reduction in the 28-day compressive strength of 78.8% for the 60 wt% RS case, which demonstrates the low volcanic ash activity of RS. On this basis, 5 wt% of phosphogypsum (PG) was added to excite the volcanic ash activity of RS, and the RS-PG-OPC ternary composite showed a delayed hydration reaction, albeit with improved flowability and improved mechanical strength at a later stage. A large amount of construction waste soil has been produced through the rapid development of urban construction, with completely decomposed granite (CDG) being the main component, which was mixed with cement and a foaming agent to produce foamed lightweight soil in the study of Tai et al. [6]. It is shown that the compressive strength was significantly affected by the investigated factors, with cement dosage being the most effective parameter, followed by CDG dosage, water/solid material (W/M) ratio, gravel particle content, and fine particle content, and the internal structure was only significantly influenced by cement dosage and W/M ratio. By characterizing the micro-pore structure, it is suggested that with the decrease in open porosity, the compressive strength showed an increasing trend. 

Apart from being the essential ingredient in concrete, cement has been used to solidify and treat toxic and harmful solid waste. Electrolytic manganese residue (EMR) produced from the manganese metal production is calcined at a high temperature and pressure and desulfurized to prepare desulfurized manganese residue (DMR), so as to minimize the serious environmental pollution and safety risks, though it is not clear whether there is a risk of heavy metal leaching from the large stockpile of DMR. Recently, Wang et al. [7] used cement as a curing agent to solidify DMR. It is reported that the flexural and compressive strengths of the cement-DMR solidified body were significantly improved by increasing the cement content to 80 mesh particle size. Meanwhile, it was found that cement can effectively solidify the soluble Mn in DMR, and the reacted products, including C-S-H gel and ettringite, form a dense network structure, which improves the strength of the solidified body.

The contributions included in this Special Issue provide a comprehensive reference for the structure characterization, property analysis, developments, and engineering application of cemented-based composites, which could inspire the investigation, industrial applications, and recycling of cement-based composites in transportation and civil engineering fields. Our Editorial Team Members are grateful to all the authors for their efforts in this Special Issue and the reviewers for their rigorous and professional support for this Special Issue.
